# Dataset comparing the effectiveness of perineal cold pack application over oral paracetamol 1000mg on postpartum perineal pain among women after spontaneous vaginal delivery in Dodoma region

**DOI:** 10.1016/j.dib.2023.109766

**Published:** 2023-11-07

**Authors:** Joyce Augustino, Fabiola Moshi, Angelina Joho, Joanes Faustine, Kihulya Mageda

**Affiliations:** a School of Nursing and Public Health, University of Dodoma, PO Box 395, Dodoma, Tanzania; b President's Office, Regional Administrative Secretary and Local Government (PORALG)

**Keywords:** Cold application, Perineal pain, Postnatal pain, Vaginal delivery

## Abstract

The data were collected from the Dodoma Regional Referral Hospital randomized clinical trial among postnatal women. The raw and analyzed data includes 228 postnatal women with the following information: age(years), marital status, education level, occupation, religion, residence, and income. It also includes the number of Antenatal Visits, perineal condition, birth weight of the newborn, and the magnitude of perineal pain at the start(time=zero), at 20,40 and 60 minutes.

The participants were randomly allocated to either an intervention or control group. The intervention group received the cold pack, while the control group received the Paracetamol 1000mg start. Data were collected using a standardized questionnaire and then analyzed using Stata™ software (StataCorp LLC, College Station, TX, US) version 14 and IBM SPSS statistics 25. The outcome was pain intensity measured using a pain rating scale at the interval of 20 minutes up to 60 minutes. The intervention's effect was estimated using an analysis of variance(repeated measure ANOVA). Omega square test was used to establish the effect size. These data will help nurse midwives in health facilities analyze data and demonstrate the effectiveness of cold packs in relieving pain instead of oral paracetamol, hence increasing scaling up its utilization.

Specifications Table

**Table:** 

Subject	Health and medical sciences
Specific subject area	Obstetrics, Midwifery and Women's Health
Data format	Raw, Analysed,
Type of data	Table, Figure
Data collection	*Data were collected using a questionnaire. The magnitude of pain was collected using a numeric rating scale. The participants were postnatal women after spontaneous vaginal delivery. The sample size was 228, of which 114 received 1000mg of oral paracetamol, and 114 received cold pack application.* All post-natal mothers who had a postpartum hemorrhage, eclampsia, shock, any medical disorders like sickle cell disease, postpartum cardiomyopathy, foetal anomaly, neonates admitted in the intensive care unit, retained placenta, consumption of alcohol or continuous drugs, 3rd or 4th-degree perineal tear, perineal hematoma, and perineal edema, were excluded.
Data source location	**Institution**: Dodoma Regional Referral Hospital **City/Town/Region**: Dodoma **Country**: Tanzania **Latitude and longitude**: Dodoma Region is located south of the equator between 6° 57′ and 3° 82′. Longitudinally, the Region is situated between 36° 26′ and 35° 26′ east of Greenwich.
Data accessibility	Repository name: Figshare Data identification number: https://doi.org/10.6084/m9.figshare.24268762.v1 Direct URL to data: https://figshare.com/articles/dataset/_b_Dataset_comparing_the_effectiveness_of_perineal_cold_pack_application_over_oral_1000mg_paracetamol_intake_in_perineal_pain_relief_among_postnatal_women_after_spontaneous_vaginal_delivery_in_Dodoma_region_b_/24268762

## 1 Value of the Data

• Despite the negative impact on a woman's daily activities, perineal pain following birth is neglected by caregivers and usually not reported by women who may consider it an expected outcome of giving birth [1]. Hence, this data is valuable to nurse midwives to analyze and advocate the utilization of cold-pack for nursing postnatal women instead of paracetamol, which sometimes exert side effect on the breastmilk for the newborn.
• Since refrigerators are well distributed in health facilities for keeping vaccines, they can produce cold packs that can benefit in relieving perineal pain among postnatal women with subsequent initiation of breastfeeding within one hour.
• The data provide a resource for leaders and policymakers to formulate effective policies for using non-pharmacological agents like cold packs to relieve perineal pain instead of pharmacological analgesics.
• The data will be valuable for training researchers in analyzing repeated measure ANOVA or using ANCOVA in experimental and observational data.
• The data provide a framework for future in-depth studies on using non-pharmacological agents in health facilities for relieving pain and triggering timely initiation of breastfeeding.
• This data further stimulates the development of a demonstration program aiming at scaling up at all health facilities, filling the gap of frequent out-of-stock pharmacological agents, and reducing the burden of out-of-pocket for mothers.

## 2 Data Description

Perineal pain after childbirth affects the woman's relationship with her child and the family [2,3]; these complications alert many research scientists to find the local application for relieving perineal pain in women after spontaneous vaginal delivery [5], [6], [7], [8]. The data presented in this article were collected from the randomized clinical trial of postnatal women who went spontaneous vaginal delivery. The raw data are demographic, obstetric characteristics, and pain level and are shared publicly in the Figshare repository [4]. The data descriptor was provided with the shared data set (Table 1). The name of the dataset in the Figshare is a coldpack_paracetamol Clinical Trial. The dataset includes the sociodemographic characteristics of the mother, such as age, marital status, education level, rural or urban residency, occupation, and sources of income. It further includes the obstetric characteristics of the mother, such as the number of pregnant(gravidity), the number of births (parity), the condition of the perineum after birth (perineum outcome), and the birth weight of the child. The data set contains measurements of the level of pain. The pain level was measured using a numeric rating scale represented by the horizontal line marked from zero to ten. It also contains the time the pain was measured, starting at time zero, 20 min, 40 min, and lasting at 60 min.

**Table 1:** Attributes and description of socio-demographic and clinical data obtained during the trial *(N=*228).

Attribute	Description
Id	Identification number of the mother
Time	Time 1,2,3,4(“1”start,”2”20minutes,”3”40minutes,”4”60minutes
score	A numeric rating scale measured a score of pain at time 1,2,3 and 4
group	Paracetamol vs cold pack
age	Age of the mother in years
maritalstats	Marital status of the mother
occupation	Occupation of the mother
educ	The education level of the mother
residence	Resident of the women, either urban or rural
numvisit	Number of antenatal visits completed by the mother while pregnant
parity	Number of births experienced by the mother
gravity	Number of pregnancies of the mother
income	The income of the family of the women
childsex	The biological sex of the child
birthweight	Birth weight of the newborn
Perineumoutcome	The perineal outcome of the mother after delivery, whether it is intact or teared
digreetare	Extent of tare of the perineum
breastfeeding	Initiation of breastfeeding of the newborn within an hour or after one hour

**Fig. 1:**
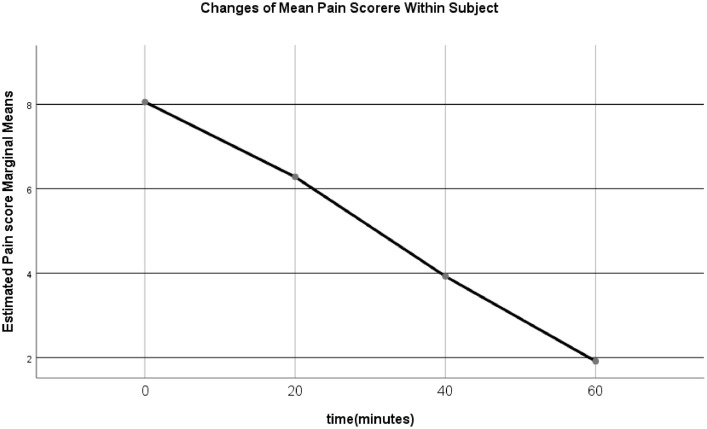
Estimated Means pain score reduction with time.

The analyzed data produces the descriptive characteristic of the pain core of the paracetamol and cold pack (Table 2); the descriptive in this table was produced by repeated measure ANOVA using IBM SPSS statistics 25 software. The repeated measure ANOVA subsequently produced Fig. 1 [4].

**Table 2:** Estimated Mean, standard deviation, within and between subjects of the study population.

*Time(minutes)*	*Group*	*Mean*	*Standard deviation*	*Total number*
0Minutes(t1)	Cold-pack	8.66	1.37	114
	Paracetamol	7.46	2.01	114
	Total	8.06	1.82	228
20minutes(t2)	Cold-pack	5.68	2.03	114
	Paracetamol	6.88	1.82	114
	Total	6.28	2.01	228
40minutes(t3)	Cold-pack	3.18	1.62	114
	Paracetamol	4.67	1.43	114
	Total	3.92	1.70	228
60minutes(t4)	Cold-pack	0.98	0.98	114
	Paracetamol	2.84	1.19	114
	Total	1.91	1.43	228

**Fig. 2:**
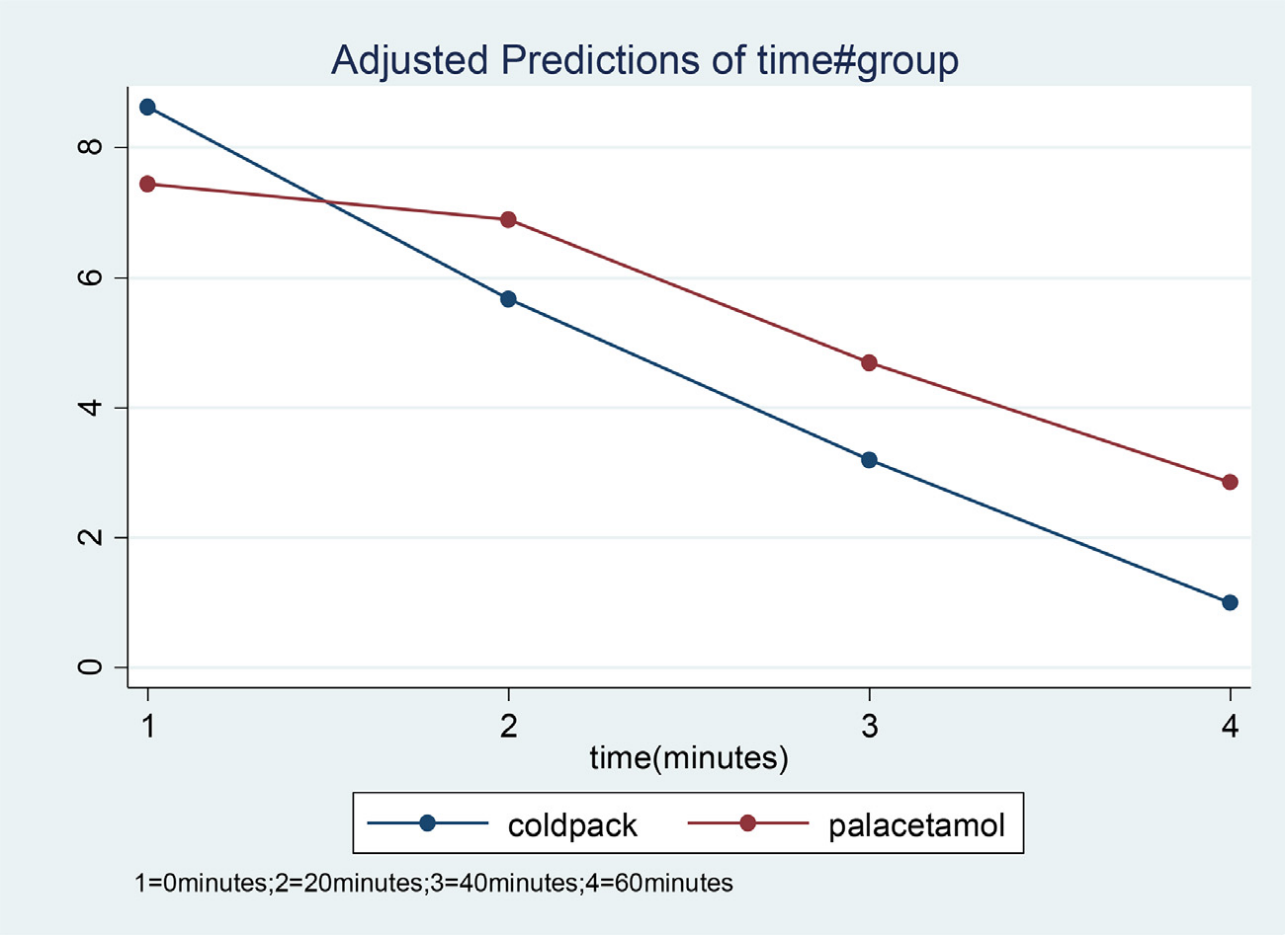
Trends in mean pain changes between paracetamol and cold pack groups to time.

Table 2 was produced as an output using repeated measure ANOVA using Stata version 14. Thus, due to a series of post-estimation commands, Table 3, Table 4, Table 5, Table 6 and Fig. 2 was produced [4].

**Table 3:** The first output of the repeated measure ANOVA.

				Obs per group:		
					min =	3
					avg =	4.0
					max =	4
						
				Wald chi2(7)	=	3725.19
Log-likelihood = −1382.0221				Prob > chi2	=	0.000
Score	Coef.	Std. error	z	P>|z|	95% conf.Interval	
group paracetamol	−1.185928	0.2282055	5.20	0.000	−1.633202	10.7386534
time						
2	−2.957858	0.1110192	−26.64	0.000	−3.175451	−2.740264
3	−5.431542	0.1404967	−38.66	0.000	−5.70691	−5.156174
4	−7.63329	0.1495089	−51.06	0.000	−7.926328	−7.340264
Group#time						
Paracetamol#2	2.413998	0.156739	15.40	0.000	2.106795	2.721201
paracetamol@3	2.685928	0.1984823	13.53	0.000	2.292782	3.074946
Paracetamol#4	3.036805	0.2112402	14.38	0.000	2.622782	3.450828
_cons	8.624524	0.1614948	53.40	0.000	8.308	8.941048

Random-effects parameters		Estimate	Std. Err.	[95% Conf. Inverval]		

Id:	empty					

Residual Unstructured						

	Var(e2)	2.963679	0.2776814	2.466482	3.561101	
	Var(e2)	3.702061	0.3467279	3.081213	4.448007	
	Var(e3)	3.412319	0.2259333	2.007765	2.898389	
	Var(e4)	1.184865	0.1109724	0.9861583	1.423609	
	Cov(e1,e2)	2.635083	0.2803297	2.085647	3.184519	
	Cov(e1,e3)	1.567611	0.2053387	1.165155	1.970068	
	Cov(e1,e4)	0.8049085	0.135066	0.5401841	1.069633	
	Cov(e2,e3)	2.344952	0.2515664	1.85189	2.838013	
	Cov(e2,e4)	1.248999	0.1614944	0.9324761	1.565522	
	Cov(e3,e4)	1.187134	0.1368107	0.9189904	1.455278	
LR test vs. linear model: chi2(9) =680.15					Prob > chi2 =0.000	

*Note:* The reported degrees of freedom assumes the null hypothesis is not on the boundary of the parameter space. If is this not true, then the reported test is conservative.

**Table 4:** Estimated degree of freedom, chi-square, and two-tailed *p*-values for the Final contrast of marginal linear prediction of the group and time.

variable	Degree of freedom	Chi-squared	*P*-values
Pain score			
Group#time	3	317.07	0.001

**Table 5:** Estimated degree of freedom, chi square, two-tailed *p*-values, contrast coefficiency, standard error, and 95% confidence interval for the group contrast.

Variables	Degree of freedom	Chi-square	-values
**Pain score**			
Time#group			
(2 VS 1) (palacetamol vs coldpack)	1	237.20	0.001
(3 VS1) (palacetamol vs coldpack)	1	183.12	0.001
(4 VS 1) (palacetamol vs coldpack)	1	206.67	0.001
Joint	3	317.07	0.001

**Pain score**	**Contrast**	**StandardError.**	**95%Confidence Interval**

Time#group			
(2 VS 1) (paracetamol vs coldpack)	2.41	0.16	2.11-2.72
(3 VS1) (paracetamol vs coldpack)	2.68	0.20	2.30- 3.07
(4 VS 1) (paracetamol vs coldpack)	3.04	0.25	2.62-3.45

**Table 6:** Estimated coefficients, degree of freedom, and 95% confidence interval for the measure of the effect size of cold-pack application on pain relief.

Time	Omega squared (%)	95% confidence interval (%)
Baseline-20 min	45	37 - 53
Baseline -40 min	38	29 - 47
Baseline-60 min	52	43 - 59

The data set can be changed to a wider format and produces results of ANCOVA as an alternative analysis of repeated measure (Table 7)

**Table 7:** shows the output of the ANCOVA.

	Number of obs = 227		R-squared = 0.6672		
	Root MSE = 1.17441		Adj R-squared = 0.6642		
Source	Partial SS	df	MS	F	Prob>F
Model	619.43602	2	309.71801	224.56	0.0000
group	263.9444	1	263.9444	191.37	0.0000
scorel	534.67016	1	534.67016	387.65	0.0000
Residual	308.95164	224	1.3792484		
Total	928.38767	226	4.10791		

MSE: Mean sum of square; SS: Sum Square; MS: Mean square; df: degree of freedom

## 3 Experimental Design, Materials and Methods

This data was collected from a randomized clinical control trial (RCCT) study involving post-natal women aged 18 to 49 allocated to the intervention and control group from a Dodoma Regional Referral Hospital postnatal ward. The number of participants in the intervention group was 114, while the control group contained 114 participants. Postnatal mothers with singleton birth, Birth weight (2500g and above), full-term, 18 to 49 years of age, and spontaneous vagina delivery (SVD) were included in the study. All post-natal mothers who had a postpartum hemorrhage, eclampsia, shock, any medical disorders like sickle, postpartum cardiomyopathy, fetal anomaly, neonates admitted in the intensive care unit, retained placenta, consumption of alcohol or continuous drugs, 3rd or 4th-degree perineal tear, perineal hematoma, and perineal edema, were excluded.

The research assistant (Nurse midwife) identified and recruited postnatal women who met the eligible criteria. The women who met the inclusion criteria and agreed to participate were included in the study by simple randomization via the closed envelope method. The participants were asked to pick any paper from the envelope and read whether it was written paracetamol (control group) or cold pack application (intervention group) as they came from the labour ward to postnatal.

The research team prepared the cold pack using examination gloves filled with water and placed it in the existing health facility refrigerators one night before the intervention. The cold pack was rapped with cotton gauze before application over the perineum to avoid direct contact with skin and fit the perineum according to the anatomy of the perineum. At the beginning of the study, the researchers collected baseline data using a standardized structured questionnaire.

Pain magnitude was evaluated by the numeric rating scale represented by a horizontal line with numerical marks from 0 (no pain) to 10 (worst imaginable pain) shown to each woman to quantify her pain sensation.

The perineal pain was self-evaluated at four time periods: the first evaluation was carried out prior to the application of the cold pack (intervention group) and paracetamol to the control group (before intervention); the second took place immediately after the application of the pack to the intervention group and paracetamol 1000mg to the control group (at 20 min); the third took place 20 min later (at 40 min); and the fourth evaluation was made once again 20 min after the third evaluation (at 60 min). Participants were followed at 20, 40, and 60 min in both intervention and control arms to assess the pain level. The dependent variable was the intensity of pain, which was measured by a pain scale known as the Numeric Rating Scale (NRS) marks from 0 (no pain) to 10 (worst imaginable pain) shown to each woman to quantify her pain scale.

Both outcomes were measured before and after the intervention in both arms. Because this was an intervention, the independent variable received either the perineal cold pack or oral paracetamol 1000mg.

Dodoma Regional Referral Hospital was selected purposively to represent the Dodoma region because pregnant women from different Districts of Dodoma Region and the nearby Region are referred.

Data were collected quantitatively using a standardized and modified structured questionnaire adopted from a previous study (East et al., 2020; Mekonnen & Shewangizaw, 2022; Senol & Aslan, 2017).

The data was entered and stored into Excel™(Microsoft Excel Worksheet (.xlsx) and analyzed using StataSE14™ (StataCorp LLC, College Station, TX, US) and IBM SPSS statistics 25 software. Descriptive statistics summarize continuous data, such as the mean, median, standard deviation, and interquartile range. One-way between-subjects comparing two groups using repeated ANOVA was done. Omega-squared was calculated to measure the effect size for planned comparison. F-tests, with their corresponding p-values, were used as improvement measures. All statistical tests were two-sided; p <0.05 was considered statistically significant. The study was open-label; the women and providers were aware of the intervention received.

## Limitations

The application and observation of the mother take one hour, the effect of reducing perineal pain, and no follow-up; this limits our dataset conclusion.

## Ethics Statement

The Institutional Ethics Clearance Board of the University of Dodoma reviewed and approved the protocol on July 04, 2023 (reference number MA:84/261/02/A/64/39). Informed consent was obtained from postnatal mothers to participate in the study; the Declaration of Helsinki carried out the study.

## CRediT Author Statement

**Joyce Kimario:** Conceptualization, Methodology, Data collection, Writing – Original draft preparation, **Kihulya Mageda:** Data Analysis, Writing – Reviewing and Editing: **Fabiola Moshi:** Conceptualization, Methodology, Supervision. **Angelina Joho:** Supervision and review: **Joanes Faustine^a^** Conceptualization and review.

## Data Availability

Dataset comparing the effectiveness of perineal cold pack application over oral paracetamol 1000mg on postpartum perineal pain among women after spontaneous vaginal delivery in Dodoma region (Original data) (Figshare)
